# Regional variation in traumatic brain injury patterns, management and mortality: a nationwide Swedish cohort study

**DOI:** 10.1007/s00701-025-06557-w

**Published:** 2025-05-08

**Authors:** Francisco Leal-Méndez, Anders Lewén, Amanda Gu, Anders Hånell, Lina Holmberg, Per Enblad, Fredrik Linder, Teodor Svedung Wettervik

**Affiliations:** 1 Department of Medical Sciences, Section of Neurosurgery, Uppsala University, 751 85 Uppsala, Sweden; 2 Department of Surgical Sciences, Section of Vascular Surgery, Uppsala University, 751 85 Uppsala, Sweden

**Keywords:** Craniotomy, Epidemiology, Neurointensive care, Outcome, Traumatic brain injury

## Abstract

**Background:**

Sweden covers a large land area, but is sparsely populated. The country is divided into six heterogenous healthcare regions, each with different geographic conditions and referral patterns when it comes to traumatic brain injury (TBI). This study aimed to explore the variation in demography, injury patterns, care pathways, management, and mortality (30 d) for TBI patients within the country.

**Methods:**

A nationwide, observational study, using data from the Swedish Trauma Registry (SweTrau) between 2018–2022, was performed. A total of 5036 TBI patients were included. Data on demography, admission status (through Glasgow Coma Scale [GCS] value at arrival at first managing hospital), injury-related variables, and mortality (30 d) were evaluated.

**Results:**

The median age was 65 years (interquartile range 46–78), and the majority of patients were male, had sustained fall-related injuries, and were conscious upon admission. Slight, but significant differences (p < 0.05) existed among the regions in these variables. In multivariate logistic regression models, the healthcare region (p < 0.05) was independently associated with patient referral to a university hospital (as compared to care at a local hospital alone), craniotomy rate, and receiving an intracranial pressure-monitoring device, after adjustment for demographic and injury variables. In similar regressions regarding mortality, specific healthcare regions (p < 0.05) were independently associated with said outcome.

**Conclusions:**

The study highlights, from a systems-level perspective, that there was a significant variation in care pathways and management among the six healthcare regions in Sweden, which might have impacted on clinical outcome. These findings call for more granular studies to understand which aspects of patient management that were particularly beneficial or detrimental for patient survival and recovery.

**Supplementary Information:**

The online version contains supplementary material available at 10.1007/s00701-025-06557-w.

## Introduction

Traumatic brain injury (TBI) is a disease with complex pathophysiology, which in moderate-to-severe cases may require both urgent and highly specialized care [2, 3, 18]. This includes evacuation of traumatic intracranial mass lesions [2, 11] and neurointensive care with multimodality monitoring [18]. The access to such care may be limited, e.g., in low-to-middle income countries with few neurosurgeons per million capita [13, 16]. Also, in more advanced healthcare systems, variations in geographical conditions and transportation logistics may affect the access to emergency neurosurgical care. The Swedish healthcare system is divided into six geographically heterogenous regions, typically with only one neurosurgical center each [11]. In regions covering larger geographical areas, but with lower population density, the distance to the university hospital with all available trauma sub-specialties may be long, and the TBI patients therefore usually undergo primary trauma surveys and initial resuscitation at a local hospital without neurosurgical expertise. In patients who present with brain herniation syndrome due to significant traumatic extracerebral lesions at the local hospital, the general surgeons locally may need to perform the hematoma evacuations if a delay in surgical timing cannot be tolerated [5, 11]. Although admittedly very uncommon internationally, this practice is established in certain Swedish regions. Most severe cases are otherwise transferred to the university hospital, while patients with milder TBI may stay at the local hospital for observation and medical management. However, in more populated, but geographically smaller healthcare regions, the distance to the university hospital may be shorter, and a greater proportion may be transferred there directly from the trauma scene providing them with immediate access to highly specialized care. Thus, differences in geographical and logistic conditions within each healthcare system may impact the level of care and expertise, which, in turn, may influence patient outcome. In an international perspective, it is also important to emphasize that even if the TBI patients reach a neurosurgical unit at a university hospital, the provided care may be heterogenous among the specialized centers, even if they, in broad terms, adhere to the general guidelines [3]. For example, in the multi-center, European CENTER-TBI cohort, it was clearly demonstrated that there were significant variations in care pathways, e.g., admission to ward vs. intensive care unit [8, 17]therapeutic intensity levels [8], and outcome that depended on the TBI center rather than demography and injury severity. These findings highlight that patient outcome can be affected by variations in geographical and logistical aspects within a healthcare region, as well as the role of local traditions and expertise of the local and university hospitals within such a region.

To further explore the differences in the organization and provided care in Sweden for different healthcare regions, we aimed to investigate any potential heterogeneity in care pathways and mortality among the six healthcare regions. We hypothesized that the great variations in geographical conditions and local traditions among the regions would result in differences in patient management and clinical outcomes.

## Materials and methods

### Patients and study design

**Fig. 1:**
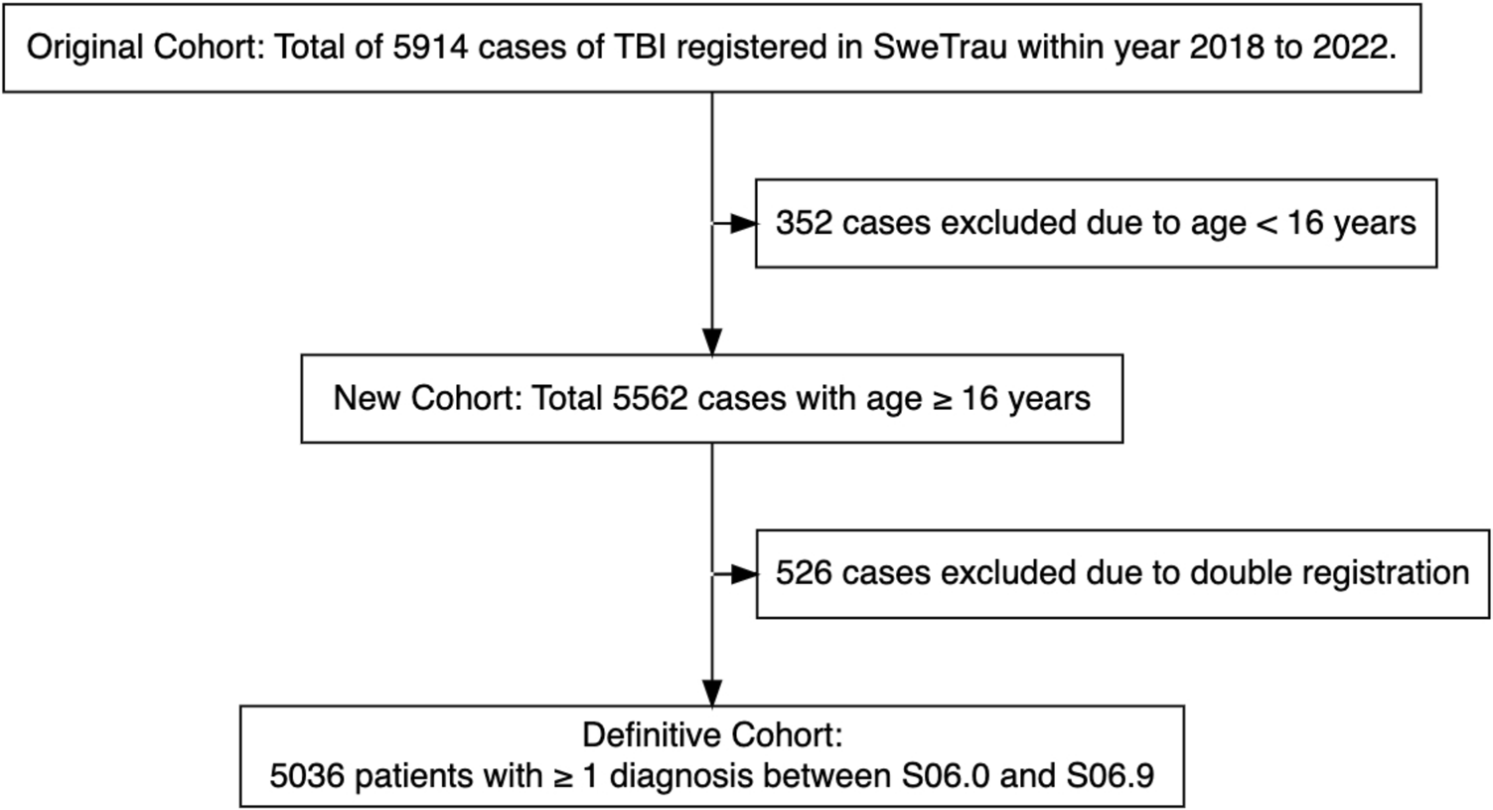
Flow chart of patient inclusion. In this study, 5036 patients over the age of 16 years in the SweTrau register with TBI (ICD-10 codes S06.0 to S06.9) who had been treated at any ICU in Sweden from the 1 st of January 2018 to the 31 st of December 2022 were included. *ICD*, International Classification of Diseases; *ICU*, Intensive Care Unit; *TBI*, Traumatic Brain Injury

This was a nationwide, multi-center study based on the Swedish Trauma Registry (SweTrau) [7]. The SweTrau registry includes trauma patients with an activated trauma call or New Injury Severity Score (NISS) > 15 [9]. In this study, data from 5914 patients of all ages registered in SweTrau who had a TBI (ICD-10 codes S06.0 to S06.9) in Sweden from the 1 st of January 2018 to the 31 st of December 2022 were extracted. Of those 5914 patients, 352 were excluded due to age below 16 years. Hereon, a cohort of 5562 cases remained. Subsequently, 526 cases of double registration were identified and excluded. Thus, the final study cohort included 5036 patients (Fig. 1).

### Structure of Swedish healthcare system and neurosurgical care in traumatic brain injury

**Fig. 2:**
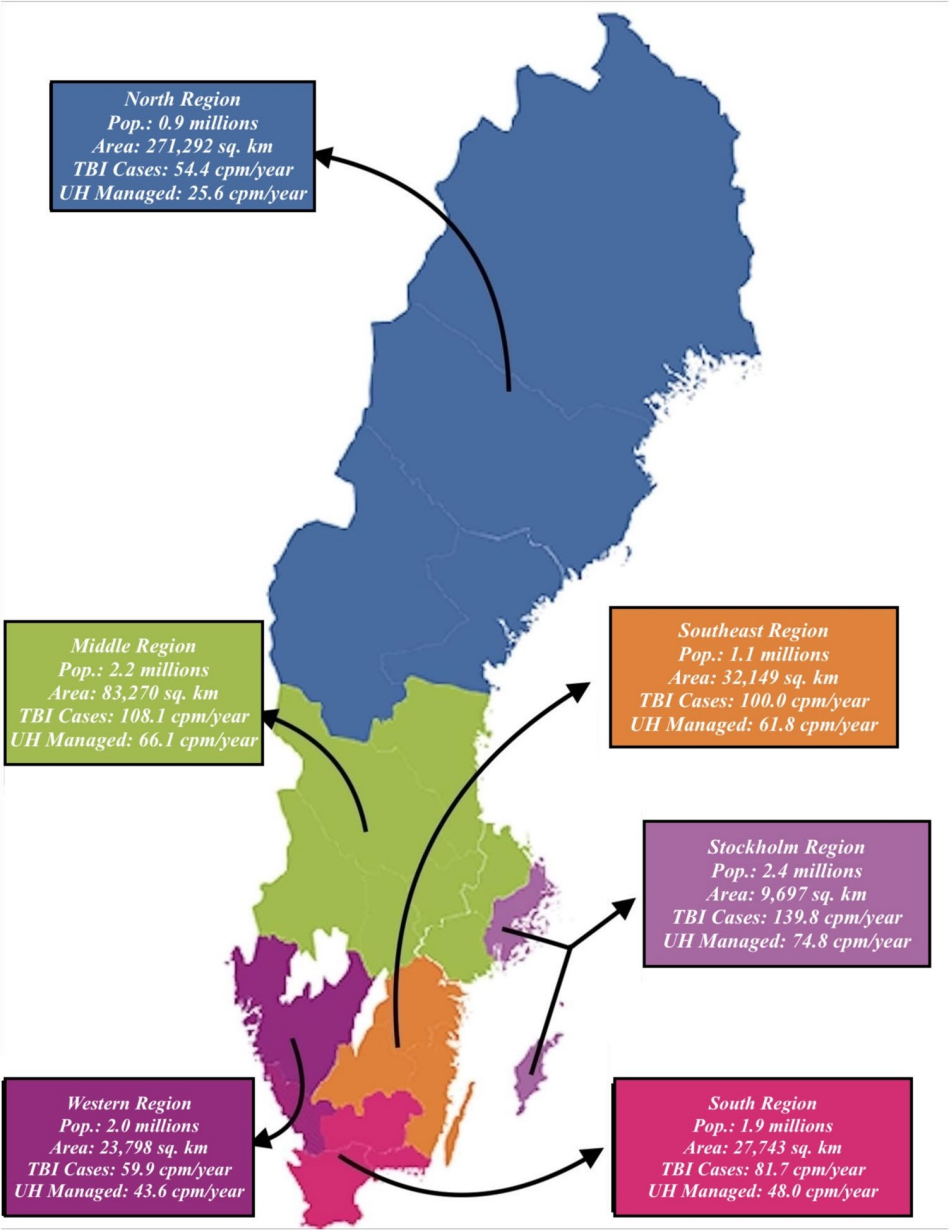
Map of Sweden divided into its six healthcare regions. *Pop*, Population; *TBI*, Traumatic brain injury; *UH*, University hospital. *Cpm/year*, Cases per million and year

As previously mentioned, the healthcare system in Sweden is divided geographically into 6 major administrative regions (Fig. 2), which differ in geographical area and population size. The Stockholm region, contains the smallest geographical area (9,697 km^2^), but the largest population (2.4 million), while the North region contains the largest geographical area (271,292 km^2^), but the smallest population (0.9 million). Each region has one University Hospital that operates as tertiary center for neurosurgical care, except the middle region which has two centers (Uppsala and Örebro, respectively, where Uppsala receives the majority of trauma patients). Furthermore, every region has several local hospitals.

All neurosurgical centers mainly adhere to the Brain Trauma Foundation guidelines of TBI regarding surgical decision-making [2] and neurointensive care [3]. General surgical management includes surgical evacuation of intracranial bleedings with mass effect. The neurointensive care is focused on optimizing brain physiology, which typically entails invasive intracranial pressure (ICP) and cerebral perfusion pressure (CPP) monitoring in unconscious patients. However, each center has their own local traditions and adaptations [1, 6, 14, 19, 21].

### Data acquisition

Data entry was performed locally at each hospital by dedicated trauma registrars, most often intensive care nurses who have completed formal training in injury coding through the Abbreviated Injury Scale (AIS) course (a standardized 2-day education program). All data were retrieved from SweTrau [7]. The main variables of interest were related to demography (age, sex), preoperative morbidities using American Society of Anesthesiologists (ASA) score [10], trauma mechanisms, injury severity (Glasgow Coma Scale [GCS], Injury Severity Score [ISS] [9], AIS head [20]), presence and type of intracranial hematomas, and neurosurgical procedures (craniotomy, ICP-monitoring). Time variables were also included (time from trauma to hospital, time from trauma to first computed tomography, and time from trauma to intervention). Outcome was evaluated as mortality 30 days post-injury.

### Statistical analysis

Continuous/ordinal variables were described as median (interquartile range [IQR]) and categorical variables as number (proportion). The Pearson’s Chi-square and Kruskal–Wallis tests were used for the statistical analyses to explore potential differences in demography, trauma mechanisms, injury severity, treatments, and outcome among the six different regions. Missing values were excluded from the analyses. These analyses were conducted for the entire cohorts of each healthcare region and for each sub-cohort of patients, treated either at the local hospital only or at the university hospital (both those who were immediately admitted there and those referred from a local hospital). To explore if there were regional variations in patient referral to the university hospital, craniotomy rate, and ICP-monitoring, respectively, a multivariate logistic regression analysis was conducted for each of these dependent variables and healthcare region, including demography (age), clinical status (GCS and ISS) and neuroradiological findings (epidural hematoma [EDH], acute subdural hematoma [ASDH], traumatic subarachnoid hemorrhage [tSAH], and contusions), as the explanatory variables. Furthermore, to explore if the healthcare region where the patient was managed influenced clinical outcome, a multivariate logistic regression with mortality as the dependent variable was conducted. In addition to the healthcare region, the same explanatory variables of demography, clinical status and neuroradiological findings were included as in the regressions above. The same regressions were conducted for each sub-cohort treated either at the local hospital alone or the university hospital to explore more specifically the extent of impact of the care at the local hospital vs the neurosurgical and neurointensive care at the university hospital on patient outcome. A p-value < 0.05 was considered statistically significant. The statistical analyses were conducted using RStudio software (RStudio 2024.04.1). All missing data was excluded from the analyses.

## Results

### Demography, trauma mechanisms, injury severity, type of injury, and treatments – entire cohort and healthcare regions

In the entire cohort (Table 1), the median age was 65 (IQR 46–78) years and most patients (68%) were male. Falls (62%) were the predominant trauma mechanism, followed by road traffic accidents (28%). The median GCS at admission was 14 (IQR 12–15), the median ISS was 17 (IQR 10–26) and the median ASA was 2 (IQR 1–3). Around half of the patients or more exhibited an ASDH, tSAH, or contusions, while EDHs were rarer (10%). A slight majority (59%) was managed at the university hospital, while 41% were only treated at the local hospital. Twelve percent required a craniotomy and 10% received ICP-monitoring. The cohort’s median number of days spent on ventilator was 2 (IQR 1–8). As demonstrated in Table 1, even if these variables were relatively similar among the regions, there were significant differences. Furthermore, there were differences in the time logistics of the trauma management among the regions (Supplementary Table 2), e.g., the median time from trauma to hospital was shorter in the Stockholm region (1.23 h) and longer in the North region (1.80 h). The study included an average of 1,007 registered patients per year. However, patient registration in 2019 (n = 988) and 2020 (n = 904) was below average due to staff allocation challenges during the COVID-19 pandemic, some regions being more affected than others. In contrast, 1,251 patients were registered in 2022, the last year analyzed in this study. Despite some variation, the overall results were still comparable to those of the year 2022 alone (see Supplementary Table 1).

**Table 1:** Demography, trauma mechanism, injury severity, type of intracranial bleedings, management, and outcome – entire cohort

Variables	Entire cohort	Stockholm	Middle	South	Western	Southeast	North	p-value
Patients, n (%)	5036 (100%)	1678 (33%)	1188 (24%)	776 (17%)	599 (11%)	550 (11%)	245 (5%)	n/a
*Demography*								
Age (years), median (IQR)	65 (46–78)	66 (48–80)	65 (45–77)	64 (45–77)	63 (44–77)	67 (46–78)	61 (43–76)	**0.001**
Sex (male/female), n (%)	3412/1624 (68%/32%)	1080/598 (64%/36%)	830/358 (70%/30%)	534/242 (69%/31%)	421/178 (70%/30%)	367/183 (67%/33%)	180/65 (73%/27%)	**0.004**
*Injury mechanisms*								
Fall, n (%)	3124 (62%)	1194 (71%)	687 (58%)	431 (56%)	345 (58%)	337 (61%)	130 (53%)	** < 0.001**
Roads, n (%)	1418 (28%)	329 (20%)	378 (32%)	259 (33%)	194 (32%)	167 (31%)	91 (37%)	
Blunt, n (%)	301 (6%)	118 (7%)	63 (5%)	52 (7%)	28 (5%)	21 (4%)	19 (8%)	
Penetrating, n (%)	42 (1%)	14 (1%)	9 (1%)	4 (1%)	8 (1%)	7 (1%)	0 (0%)	
Explosion, n (%)	6 (0%)	2 (0%)	1 (0%)	3 (0%)	0 (0%)	0 (0%)	0 (0%)	
Other/unknown, n (%)	145 (3%)	21 (1%)	50 (4%)	27 (3%)	24 (4%)	18 (3%)	5 (2%)	
*Injury severity*								
GCS at admission, median (IQR)	14 (12–15)	14 (13–15)	14 (11–15)	14 (10–15)	14 (12–15)	14 (11–15)	14 (13–15)	** < 0.001**
AIS head, median (IQR)	3 (2–3)	3 (2–3)	3 (2–3)	3 (2–3)	3 (2–3)	3 (2–4)	3 (2–3)	** < 0.001**
ISS, median (IQR)	17 (10–26)	17 (10–25)	17 (11–25)	18 (13–26)	17 (12–26)	20 (14–25)	17 (10–25)	** < 0.001**
ASA score, median (IQR)	2 (1–3)	2 (1–3)	2 (1–3)	2 (1–2)	2 (1–3)	2 (1–3)	2 (1–3)	** < 0.001**
Epidural hematoma (yes), n (%)	525 (10%)	151 (9%)	114 (10%)	114 (15%)	53 (9%)	63 (11%)	30 (12%)	** < 0.001**
Acute subdural hematoma (yes), n (%)	3652 (73%)	1277 (76%)	854 (72%)	545 (70%)	409 (68%)	398 (72%)	169 (69%)	**0.001**
Traumatic subarachnoid hemorrhage (yes), n (%)	2416 (48%)	845 (50%)	546 (46%)	382 (49%)	279 (47%)	224 (41%)	140 (57%)	** < 0.001**
Contusion, n (%)	2475 (49%)	875 (52%)	533 (45%)	411 (53%)	309 (52%)	239 (43%)	108 (44%)	** < 0.001**
*Management*								
Managed at university vs regional hospital alone, n (%)*	2972 (59%)/2061 (41%)	898 (56%)/778 (44%)	727 (65%)/461 (35%)	456 (64%)/320 (36%)	436 (74%)/163 (26%)	340 (66%)/210 (34%)	115 (51%)/129 (49%)	** < 0.001**
Craniotomy (yes), n (%)	587 (12%)	206 (12%)	143 (12%)	109 (14%)	51 (9%)	59 (11%)	19 (9%)	**0.011**
ICP-monitoring (yes), n (%)	481 (10%)	132 (8%)	172 (14%)	80 (10%)	33 (5%)	29 (5%)	35 (14%)	** < 0.001**
Days on ventilator, median (IQR)	2 (1–8)	3 (1–11)	2 (1–7)	2 (1–7)	2 (1–6)	2 (1–6)	5 (1–10)	***0.002***
*Outcome*								
Mortality, n (%)	923 (18%)	250 (15%)	207 (17%)	184 (24%)	132 (22%)	117 (21%)	33 (13%)	** < 0.001**

Missing data: Age (n = 0), Sex (n = 0), Injury Mechanism (n = 0), Management at university vs. Regional hospital alone (n = 3), GCS at admission (n = 878), ASA (n = 49), Mortality (n = 73)

AIS = Abbreviated Injury Scale, ASA = American Society of Anesthesiologists scale. GCS = Glasgow Coma Scale. ICP = Intracranial Pressure, ISS = Injury Severity Score. IQR = Interquartile Range

### University hospital care in the six healthcare regions – differences and predictors

In the university hospital cohort (Supplementary Table 3), the median age was 59 (IQR 39–73) years, and they were predominantly male (72%). The most prevalent trauma mechanism was falls (56%), followed by road traffic accidents (31%). For GCS at admission, a median of 14 (IQR 11–15) was observed. The median ISS was 21 (IQR 14–26) and ASA showed a median of 2 (IQR 1–3). Regarding intracranial lesion type over half of patients exhibited ASDH (71%), tSAH (52%) and contusions (56%) but EDH was rarer (14%). The median number of days spent on ventilator was 3 (IQR 1–9). In brief, this cohort was younger, exhibited worse systemic and cerebral injuries and required longer assisted ventilation therapy than the cohort treated at the local hospitals (Supplementary Table 4). The northern region had the lowest rate of patients treated at the university hospital (51%), while the Western region had the highest rate (74%) (Table 1). In a multivariate logistic regression (Table 2), the healthcare region was independently associated with being treated at a university hospital after adjustment for demographic and injury-related variables. In that regression, Stockholm and the Western region had the lowest thresholds to treat the patients at the university hospital.

**Table 2:** Predictors of treatment at the university hospital, craniotomy and ICP-monitoring – logistic regression for the entire cohort

	*University hospital management*		*Craniotomy*		*ICP-monitoring*	
*Variables*	*OR (95% CI)*	*p-value*	*OR (95% CI)*	*p-value*	*OR (95% CI)*	*p-value*
Age	0.98 (0.98–0.99)	** < *0.001***	0.98 (0.97–0.99)	** < *0.001***	0.97 (0.96–0.98)	** < *0.001***
GCS	1.01 (0.99–1.03)	0.285	0.97 (0.94–1.01)	0.171	0.85 (0.81–0.88)	** < *0.001***
ISS	1.04 (1.03–1.05)	** < *0.001***	1.07 (1.05–1.08)	** < *0.001***	1.06 (1.03–1.07)	** < *0.001***
ASA	1.21 (1.10–1.33)	** < *0.001***	1.30 (1.07–1.58)	***0.008***	1.51 (1.20–1.90)	** < *0.001***
EDH	1.64 (1.29–2.08)	** < *0.001***	1.52 (0.99–2.28)	***0.050***	2.59 (1.67–3.94)	** < *0.001***
ASDH	1.18 (0.99–1.41)	0.062	3.24 (2.16–4.97)	** < *0.001***	2.13 (1.43–3.23)	** < *0.001***
tSAH	1.31 (1.14–1.51)	** < *0.001***	0.79 (0.59–1.05)	0,105	1.78 (1.24–2.55)	***0.002***
Contusion	1.86 (1.59–2.18)	** < *0.001***	1.21 (0.90–1.63)	0.214	2.87 (1.99–4.19)	** < *0.001***
Stockholm Region	1.00 (Reference)	-	1.00 (Reference)	-	1.00 (Reference)	-
Middle Region	0.74 (0.62–0.89)	***0.001***	1.29 (0.92–1.82)	0.142	1.86 (1.24–2.82)	***0.003***
South Region	0.42 (0.33–0.52)	** < *0.001***	0.76 (0.48–1.16)	0.207	0.60 (0.33–1.04)	0.075
Western Region	1.94 (1.56–2.41)	** < *0.001***	0.56 (0.32–0.92)	***0.027***	0.65 (0.35–1.17)	0.165
Southeast Region	0.80 (0.63–1.02)	0.076	0.96 (0.59–1.53)	0.880	0.55 (0.24–1.12)	0.122
North Region	0.42 (0.12–0.30)	** < *0.001***	0.40 (0.13–0.95)	0.061	1.69 (0.78–3.39)	0.157

UH Management: AIC = 4908.6; *r*^2^ = 0.10; AUROC (95% CI) = 0.70 (0.67–0.73)

Craniotomy: AIC = 1058.1; *r*^*2*^ = 0.40; AUROC (95% CI) = 0.77 (0.72–0.83)

ICP-monitoring: AIC = 2286.8; *r*^*2*^ = 0.22; AUROC (95% CI) = 0.84 (0.79–0.89)

*AIC*, Akaike Information Criteria; *ASA*, American Society of Anesthesiologists scale; *ASDH*, Acute Subdural Hematoma; *AUROC*, Area Receiver Operating Characteristics Curve; *CI*, Confidence Interval; *EDH*, Epidural Hematoma; *GCS*, Glasgow Coma Scale; *ICP*, Intracranial Pressure; *ISS*, Injury severity score; *OR*, Odds Ratio; *tSAH*, Traumatic subarachnoid hemorrhage

The rate of craniotomy was 12% (n = 587, Table 1) for the entire cohort, of these 96% (n = 566) were performed at university hospitals and 4% (n = 21) at local hospitals (12 of these were later transferred to a university hospital). Craniotomy rates differed significantly among the regions, with the highest rate in the South region (14%) and the lowest rate in the Western and North regions (9%). After adjustment for demographic and injury-related variables in a logistic regression, the rate of craniotomy was significantly associated with lower odds in the Western region as compared with Stockholm (Table 2).

ICP-monitoring was performed in 16% of patients treated at university hospitals, but was never done at local hospitals (Table 1 and Supplementary Table 3). The rate of ICP-monitoring was lower in the Western (7%) and higher in the North region (30%) (Supplementary Table 3). After adjustment for differences in demography and injury-related variables in a logistic regression (Table 2), the healthcare region was significantly associated with the rate of ICP-monitoring, particularly it was more common in the Middle region (OR = 1.86, p = 0.003).

### Clinical outcome – entire cohort and health care regions

In the entire cohort (Table 1), the mortality rate 30 days after trauma was 18%. The rate was slightly higher in the South region (24%) and lower in the North region (13%) (Table 1). In the sub-cohort of patients admitted to a university hospital (Supplementary Table 3) mortality rate was lower in the North region (8%) and higher in the Western (21%) region. Among patients treated exclusively at a local hospital, the highest mortality rate was in the Southeast region (33%) and the lowest in the Stockholm region (13%) (see Supplementary Table 4). In the sub-cohort of university managed patients (Supplementary Table 3), the overall mortality rate was lower (16%) compared to the cohort of local hospital managed patients (Supplementary Table 4), which had an overall mortality rate of 22%.

In multivariate logistic regressions with mortality as the dependent variable (Table 3), the healthcare region was, in all cases except in the North region, independently associated with a slightly higher risk of mortality compared to Stockholm. This was particularly evident in the South region. In subsequent analyses, the same pattern persisted across all regions in the sub-cohort treated exclusively at a local hospital, albeit with less cases of statistical significance (Table 3). Conversely, in the university hospital cohort (Table 3), a statistically significant higher mortality was observed only in the South region.

**Table 3:** Logistic regression analysis for mortality as the dependent variable in the entire cohort, patients treated at university hospitals, and those treated at local hospitals

	Entire Cohort		University Hospital		Local Hospital	
Variable	OR (CI 95%)	P value	OR (CI 95%)	P-value	OR (CI 95%)	P value
Age (years)	1.07 (1.06–1.08)	** < *0.001***	1.07 (1.05–1.08)	** < *0.001***	1.07 (1.06–1.09)	** < *0.001***
GCS	0.71 (0.68–0.72)	** < *0.001***	0.74 (0.71–0.77)	** < *0.001***	0.65 (0.61–0.68)	** < *0.001***
ISS	1.08 (1.07–1.10)	** < *0.001***	1.08 (1.06–1.10)	** < *0.001***	1.11 (1.09–1.13)	** < *0.001***
ASA	1.82 (1.55–2.14)	** < *0.001***	1.84 (1.48–2.31)	** < 0.001**	1.93 (1.49–2.50)	** < *0.001***
EDH	0.83 (0.51–1.30)	0.420	0.73 (0.39–1.31)	0.304	1.39 (0.63–2.93)	0.402
ASDH	1.14 (0.82–1.58)	0.443	1.17 (0.75–1.83)	0.487	1.33 (0.80–2.24)	0.277
tSAH	1.24 (0.98–1.56)	0.072	1.54 (1.09–2.17)	***0.014***	0.99 (0.71–1.39)	0.985
Contusion	0.85 (0.66–1.09)	0.221	1.07 (0.74–1.53)	0.732	0.83 (0.56–1.22)	0.345
Stockholm Region	1.00 (Reference)	-	1.00 (Reference)	-	1.00 (Reference)	-
‍Middle Region	1.41 (1.04–1.91)	***0.028***	1.09 (0.69–1.71)	0.719	1.86 (1.19–2.88)	***0.006***
South Region	2.33 (1.68–3.23)	** < *0.001***	1.79 (1.09–2.91)	***0.019***	2.82 (1.76–4.52)	** < *0.001***
Western Region	1.55 (1.06–2.24)	***0.022***	1.44 (0.89–2.31)	0.136	2.24 (1.11–4.41)	***0.021***
Southeast Region	1.50 (1.02–2.18)	***0.038***	1.42 (0.81–2.46)	0.219	1.33 (0.74–2.35)	0.329
North Region	0.94 (0.49–1.72)	*0.849*	0.41 (0.12–1.17)	0.120	1.37 (0.60–2.97)	0.432

Mortality for entire cohort: AIC = 2131.1; *r*^2^ = 0.43; AUROC (95% CI) = 0.91 (0.89–0.93)

Mortality for University Hospital: AIC = 1058.1; *r*^2^ = 0.40; AUROC (95% CI) = 0.91 (0.88–0.94)

Mortality for Local Hospital: AIC = 1021.9; *r*^2^ = 0.48; AUROC (95% CI) = 0.92 (0.90–0.95)

Bold and italics indicate statistical significance

*AIC*, Akaike Information Criteria; *ASA*, American Society of Anesthesiologists scale; *ASDH*, Acute Subdural Hematoma; *AUROC*, Area Receiver Operating Characteristics Curve; *CI*, Confidence Interval; *EDH*, Epidural Hematoma; *GCS*, Glasgow Coma Scale; *ISS*, Injury severity score; *OR*, Odds Ratio; *tSAH*, Traumatic subarachnoid hemorrhage

## Discussion

The detailed results of this registry study should be interpreted with caution, especially the outcome results since the measured mortality may have been biased by several uncontrolled confounders and no data of long-term and functional outcome results were available. However, with this in mind, significant variations were found in this nationwide registry study of 5036 TBI patients, regarding demography, trauma mechanisms, type of injury, and injury severity among the six healthcare regions. Even after adjusting for such variations, there were significant differences in the care pathways for each region, as the threshold to admit the patient to a university hospital, perform a craniotomy, and insert an ICP-monitor depended on where the patient was treated. Furthermore, even after adjusting for the differences in demography and injury variables, the healthcare region was found to be independently associated with 30-day mortality. Under all circumstances, the study highlights that there are important variations in the decision-making of care pathways and treatments among the regions that may have impacted on patient outcome. Thus, even if geography and population patterns cannot be changed, there is a need to proceed with more granular clinical, radiological, and treatment studies to determine the specific reasons explaining these regional discrepancies in greater detail.

Looking more in detail at the results, the entire TBI cohort of this study was mostly based on sen ior, male adults who had sustained a head injury after a fall and suffered from a mild-to-moderate TBI. This finding in the Swedish TBI population seems compatible with other modern TBI cohorts from Europe [17]. The demographic and injury variables differed significantly among the healthcare regions, as expected, however, the general TBI pattern as described above seemed consistent in all Swedish regions.

Since the patient cohorts differed to some extent in demography and injury-related variables, it was expected to find a variation in the extent of referral to the university hospital and the rate of neurosurgical procedures including craniotomy and ICP-monitoring. It was also found that the healthcare region remained independently associated with these management variables after adjusting for such potential confounders. Particularly, the more densely populated regions with relatively smaller geographical areas, Stockholm and Western, were more prone to admit their patients to the university hospitals compared to the other regions. This finding is probably related to the geographical characteristics, in particular the distance from the trauma scene to the university hospital, which may be considerably longer in the more sparsely populated regions. It is likely in those regions that initial management according to Advanced Trauma Life Support (ATLS) at the local hospital was more feasible and after resuscitation and gaining more information about the patient, a more selected process was made regarding if the patients needed a transportation to the university hospital. In addition, other factors more difficult to characterize most likely also contributed to the variation in the rate of patient referral to a university hospital. Such factors may include, but are not limited to local staff expertise in TBI management, both surgical and conservative within the ICU, as well as local treatment traditions. Otherwise, as expected, younger age and worse systemic and brain injury were independent risk factors of being referred to a university hospital.

Regarding differences in neurosurgical procedures, the rate of craniotomy was overall comparable among the regions after adjustment for demography and injury severity-related variables, but slightly lower in the Western region. This finding may reflect a higher threshold to proceed with such surgery in that region. However, it cannot be excluded that we were unable to adjust for some potential confounding variables, such as the extent of mass effect of the traumatic lesions or suboptimal registration in this database. Regarding the rate of ICP-monitoring, this may partly be related to patient transfer logistics, i.e., directly to a university hospital vs. local hospital first. In the former case, such as in Stockholm, a greater proportion of patients were immediately transferred to the university hospital, and there it may be a higher inclination to proceed with such invasive monitoring when the patients were already there. However, still the highest rate of ICP-monitoring was in the Middle region, which is characterized by a large geographical area in which many of the patients are managed at local hospitals. This finding highlights the role of institutional policy over geographical aspects regarding the decision to proceed with such monitoring.

Regarding the clinical outcome, which must be interpreted with caution, after adjusting for potential demographic and injury confounders, it was found that the healthcare region was significantly associated with the rate of 30-day mortality. The association between outcome and healthcare region were found both in the sub-cohort treated at a local hospital and those managed at a university hospital (Supplementary Tables 3 and 4). Interestingly, the inter-center differences observed here appear smaller than those reported in studies like CENTER-TBI [8], although key differences in cohort characteristics such as a younger median age (49 vs. 65), higher ISS (29 vs. 17) and a higher rate of severe TBI cases in the CENTER-TBI study limit direct comparison. The mortality also seems to be slightly lower than in studies from other Nordic countries [12, 15] who report in-hospital fatality rate of as high as 67% for patient managed in local hospitals and 36% for patients managed at regional trauma centers with 84% and 73% respectively at 6 months follow-up. However, meaningful comparisons are challenging due to differences among the cohorts (e.g., injury severity). Although we cannot adjust for all potential confounding variables in demography and injury severity, these findings indicate that differences in patient management among the regions and/or judgement of futility might have had an impact on mortality. One contributing aspect may be time from trauma to the hospital and the first interventions. Interestingly, regions other than Stockholm (which had the shortest lead times) were able to compensate for longer distances to neurosurgical care at university hospitals to some extent. This occurred particularly in the Middle region, where neurosurgically educated general surgeons at local hospitals performed emergency neurosurgery in selected cases [11].

Other important factors that might contribute to the variation in outcome could be the patient selection for neurosurgical treatments and the timing of such procedures. Avoiding to operate on certain cohorts, e.g., old and fragile patients, due to poor prognosis may occasionally become self-fulfilling prophecies. The timing of surgical procedures may also be highly variable and delaying treatment until the patients are in a poor condition and already have suffered from secondary brain injury could be detrimental. We have no such data, but speculate that these aspects could have influenced the between-center effect on mortality. Furthermore, since the introduction of neurointensive care with structured protocols of monitoring and management to improve brain physiology and avoid secondary brain injury, several studies have consistently shown improved survival and rate of functional recovery [4]. However, less is known about the specific aspects and treatment targets in this concept that contribute to improved outcomes. Consequently, although most centers in general adhere to the Brain Trauma Foundation guidelines, local interpretations and adaptations seem to predispose for great variations in protocols and therapeutic intensity levels. Even in a small country like Sweden, there are major differences in the extent of neuromonitoring and the target thresholds of such variables. For example, some centers [1, 6, 14, 19] adhere more to the Lund concept, focused on avoiding cerebral hyperperfusion by tolerating a lower CPP, generous administration of colloid fluids, and avoidance of vasopressors, while other centers do not follow that concept [4]. Again, although we lack data to directly confirm these assumptions, we propose them as plausible explanations that warrant investigation in future studies. Lastly, there were also differences in outcome among the healthcare regions for those treated at the local hospitals, highlighting that there may also be important variations in the management of these patients.

### Methodological considerations

The study had many strengths. Particularly, it was based on a nationwide registry with relatively good coverage of trauma patients [7], a large cohort size, and detailed demographic, injury, and outcome data. There were also some limitations. First, it is important to note that during the COVID-19 pandemic, a clear decline in registration during years 2019 and 2020 was observed. Although this issue affected the entire country, it appeared more pronounced in the Western, Southeast, and North regions (data not illustrated). However, the overall results were still comparable to those of the year 2022 alone. Second, data were missing under some circumstances, both due to under-registration as previously mentioned, but also regarding some variables, such as time from trauma to intervention, which reduces the reliability and introduces some bias. Third, although the registry provided extensive clinical data, some important variables were not available, such as the rate of unreactive pupil(s), the extent of mass effect of intracranial lesions or whether the patients was on anticoagulant/antiplatelet medication. Fourth, the only reliable outcome measure that allowed for between-center comparisons was mortality within 30 days after trauma but the registry does not provide relevant information such as cause of death or even the timing of it beyond that point. However, a further limitation is that we lacked data on mortality at earlier time points, particularly prehospital deaths, as patients who died before hospital admission were not registered in the dataset. Evaluating the long-term functional outcome of patients was not possible, which limits our grasp of a more granular outcome as it encases it to a pure “all or nothing” scenario. At the same time, the advantage of a short-term outcome measure is that it is not confounded by the extent of rehabilitation care and reintegration to society. Fifth, while these results are limited to the Swedish setting with a limited external validity for e.g., Asia or Africa, they highlight important systems-level-perspective of TBI care, i.e., the implications of geographical and logistical conditions as well as local traditions and expertise on management strategies and patient outcome. Sixth, we accounted for the presence and regional variation of mild TBI cases when analyzing the outcome measures of care trajectories and mortality by adjusting for injury severity (GCS) in the multivariable regression models of these dependent variables. Seventh, only 44.3% (39/88) of trauma patients eligible for inclusion were registered in SweTrau; however, none of the missed cases were severely injured (NISS > 15) or had activated the highest level of trauma call (Trauma Alert). All missed patients were classified as Level 2 activations and were evenly distributed across the validated hospitals. Given that case completeness was 100% for patients with NISS > 15 and/or Level 1 activation—including those with a GCS ≤ 13—this suggests that the registry captures the most clinically relevant subset of trauma cases [7]. Lastly, the number of available beds in the intensive care units may have varied among regions and could have impacted on the care trajectories, however, we had no such data and, therefore, could no investigate the impact of this variable.

## Conclusions

In this nationwide registry study, the TBI panorama in terms of demography, trauma mechanisms, and injury severity differed significantly among the six healthcare regions in Sweden. However, of interest, there were differences in the care pathways of for each region, as the threshold to admit the patient to a university hospital, perform a craniotomy, and insert an ICP-monitor were significantly and independently related to where the patient was treated. Furthermore, even after adjusting for many of the differences in demography and injury variables, the healthcare region was found to be independently associated with mortality, both for patients only managed at local hospitals and those treated at the university hospitals, even if these findings should be interpreted with caution. The study highlights that there are important variations in the decision-making of care pathways and management among the regions that may have influenced patient outcome. Our findings warrant more detailed clinical, radiological, and treatments studies to elucidate the specific aspects that could explain these regional variations in TBI care and outcome.

## Supplementary Information

Below is the link to the electronic supplementary material.

Supplementary file1 (DOCX 45 kb)

## Data Availability

The data is available upon reasonable request.

## Abbreviations

*AIC*: Akaike Information Criteria
*AIS*: Abbreviated Injury Score
*ASA*: American Society of Anesthesiologists
*ASDH*: Acute Subdural Hematoma
*ATLS*: Advanced Trauma Life Support
*AUROC*: Area Under the Receiver Operating Characteristics Curve
*CI*: Confidence Interval
*CPM*: Cases Per Million
*EDH*: Epidural Hematoma
*GCS*: Glasgow Coma Scale
*ICD*: International Classification of Diseases
*ICP*: Intracranial Pressure.
*ICUs*: Intensive Care Units
*ISS*: Injury Severity Score
*IQR*: Interquartile Range
*NISS*: New Injury Severity Score
*OR*: Odds Ratio
*SweTrau*: Swedish Trauma Registry
*UH*: University Hospital
*TBI*: Traumatic Brain Injury
