# Supplementary material for: Regional variation in traumatic brain injury patterns, management and mortality: a nationwide Swedish cohort study
Source: Acta Neurochir (Wien). 2025 May 8;167(1):134. doi: 10.1007/s00701-025-06557-w (PMC12062049; doi:10.1007/s00701-025-06557-w)
Supplement: Supplementary file 1 — Supplementary file1 (DOCX 45 kb) [file 701_2025_6557_MOESM1_ESM.docx]

**Supplementary Table 1**. Demography, trauma mechanism, injury severity, type of intracranial bleedings, management, and outcome – Entire cohort – Period 2018 – 2022 vs. year 2022 alone

|  | Entire cohort | | Stockholm | | Middle | | South | | Western | | Southeast | | North | |
| --- | --- | --- | --- | --- | --- | --- | --- | --- | --- | --- | --- | --- | --- | --- |
| **Variables / Years** | 2018 – 2022 | 2022 alone | 2018 – 2022 | 2022 alone | 2018 – 2022 | 2022 alone | 2018 – 2022 | 2022 alone | 2018 – 2022 | 2022 alone | 2018 – 2022 | 2022 alone | 2018 – 2022 | 2022 alone |
| Patients (2018-2022), n (%) | 5036 (100%) | 1251 (100%) | 1678 (33%) | 442 (26%) | 1188 (24%) | 280 (24%) | 776 (17%) | 181 (23%) | 599 (11%) | 130 (22%) | 550 (11%) | 128 (23%) | 245 (5%) | 90 (7%) |
| *Demography* | | | | | | | | | | | | | | |
| Age (years), median (IQR) | 65 (46-78) | 67 (48 – 80) | 66 (48-80) | 71 (53 - 82) | 65 (45-77) | 68 (47 - 79) | 64 (45-77) | 66 (51 - 81) | 63 (44-77) | 60 (44 - 77) | 67 (46-78) | 65 (42 - 78) | 61 (43-76) | 52 (36 - 73) |
| Sex (male/female), n (%) | 3412/1624 (68%/32%) | 837/414 (67%/33%) | 1080/598 (64%/36%) | 265 (60%)/177 (40%) | 830/358 (70%/30%) | 194 (69%)/86 (31%) | 534/242 (69%/31%) | 120 (66%)/ 61 (34%) | 421/178 (70%/30%) | 92 (71%)/ 38 (29%) | 367/183 (67%/33%) | 98 (77%)/30 (23%) | 180/65 (73%/27%) | 68 (76%)/22 (24%) |
| *Injury Mechanism* | | | | | | | | | | | | | | |
| Fall, n (%) | 3124 (62%) | 795 (64%) | 1194 (71%) | 324 (73%) | 687 (58%) | 168 (60%) | 431 (56%) | 107 (59%) | 345 (58%) | 73 (55%) | 337 (61%) | 78 (61%) | 130 (53%) | 45 (50%) |
| Roads, n (%) | 1418 (28%) | 342 (27%) | 329 (20%) | 82 (19%) | 378 (32%) | 85 (30%) | 259 (33%) | 59 (33%) | 194 (32%) | 45 (35%) | 167 (31%) | 37 (29%) | 91 (37%) | 34 (38%) |
| Blunt, n (%) | 301 (6%) | 72 (6%) | 118 (7%) | 26 (6%) | 63 (5%) | 13 (5%) | 52 (7%) | 10 (6%) | 28 (5%) | 9 (7%) | 21 (4%) | 5 (4%) | 19 (8%) | 9 (10%) |
| Penetrating, n (%) | 42 (1%) | 12 (1%) | 14 (1%) | 4 (1%) | 9 (1%) | 3 (1%) | 4 (1%) | 3 (2%) | 8 (1%) | 1 (1%) | 7 (1%) | 1 (1%) | 0 (0%) | 0 (0%) |
| Explosion, n (%) | 6 (0%) | 1 (0%) | 2 (0%) | 0 (0%) | 1 (0%) | 0 (0%) | 3 (0%) | 1 (0%) | 0 (0%) | 0 (0%) | 0 (0%) | 0 (0%) | 0 (0%) | 0 (0%) |
| Other/unknown, n (%) | 145 (3%) | 29 (2%) | 21 (1%) | 6 (1%) | 50 (4%) | 11 (4%) | 27 (3%) | 1 (0%) | 24 (4%) | 2 (2%) | 18 (3%) | 7 (5%) | 5 (2%) | 2 (2%) |
| *Injury Severity* | | | | | | | | | | | | | | |
| GCS at admission, median (IQR) | 14 (12-15) | 14 (12-15) | 14 (13-15) | 14 (13 - 15) | 14 (11-15) | 14 (11 - 15) | 14 (10-15) | 14 (11 - 15) | 14 (12-15) | 14 (8 - 15) | 14 (11-15) | 14 (8 - 15) | 14 (13-15) | 15 (13 - 15) |
| AIS head, median (IQR) | 3 (2-3) | 3 (2- 3) | 3 (2-3) | 3 (2-3) | 3 (2-3) | 3 (2 - 3) | 3 (2-3) | 3 (2 - 3) | 3 (2-3) | 3 (2 - 3) | 3 (2-4) | 3 (2 - 3) | 3 (2-3) | 3 (2 - 3) |
| ISS, median (IQR) | 17 (10-26) | 17 (10 -25) | 17 (10-25) | 14 (10 - 22) | 17 (11-25) | 17 (13 - 25) | 18 (13-26) | 17 (10 - 25) | 17 (12-26) | 17 (11 - 26) | 20 (14-25) | 17 (13 - 25) | 17 (10-25) | 19 (11 - 26) |
| ASA score, median (IQR) | 2 (1 – 3) | 2 (1-3) | 2 (1 – 3) | 2 (2 - 3) | 2 (1 – 3) | 2 (1 - 3) | 2 (1 – 2 ) | 2 (1 - 3) | 2 (1 – 3) | 2 (1 - 2) | 2 (1 – 3) | 2 (1 - 3) | 2 (1 – 3) | 2 (1 - 3) |
| *Injury Type* | | | | | | | | | | | | | | |
| Epidural hematoma (yes), n (%) | 525 (10%) | 119 (10%) | 151 (9%) | 28 (6%) | 114 (10%) | 29 (10%) | 114 (15%) | 24 (13%) | 53 (9%) | 12 (9%) | 63 (11%) | 15 (12%) | 30 (12%) | 11 (12%) |
| Acute subdural hematoma (yes), n (%) | 3652  (73%) | 931 (74%) | 1277 (76%) | 343 (78%) | 854 (72%) | 206 (74%) | 545 (70%) | 130 (72%) | 409 (68%) | 94 (72%) | 398 (72%) | 99 (77%) | 169 (69%) | 59 (66%) |
| Traumatic subarachnoid hemorrhage (yes), n (%) | 2416 (48%) | 615 (49%) | 845 (50%) | 219 (50%) | 546 (46%) | 134 (48%) | 382 (49%) | 95 (52%) | 279 (47%) | 65 (50%) | 224 (41%) | 51 (40%) | 140 (57%) | 51 (57%) |
| Contusion, n (%) | 2475 (49%) | 602 (48%) | 875 (52%) | 218 (49%) | 533 (45%) | 123 (44%) | 411 (53%) | 95 (52%) | 309 (52%) | 64 (48%) | 239 (43%) | 53 (41%) | 108 (44%) | 50 (56%) |
| *Logistics* | | | | | | | | | | | | | | |
| Time from trauma to Hospital (hours), median (IQR) | 1.30 (0.85 – 3.43) | 1.45 (0.92 – 8.69) | 1.28 (0.86 – 2.81) | 1.30 (0.87 – 3.89) | 1.38 (0.85 – 8.52) | 1.70 (0.93 – 12.34) | 1.16 (0.78 – 4.09) | 1.50 (0.95 – 10.42) | 1.43 (1.00 – 4.60) | 1.23 (0.90 – 2.32) | 1.13 (0.78 – 1.83) | 1-58 (0.90 - 18.78) | 1.62 (0.78 – 6.00) | 2.36 (1.03 - 7.95) |
| Time from trauma to CT (hours), median (IQR) | 2.95 (1.78-7.00) | 2.90 (1.65-10.40) | 4.11 (2.18-7.47) | 3.31 (1.65-7.37) | 2.84 (1.71 – 10.02) | 3.01 (1.83-14.33) | 2.40 (1.50-6.27) | 2.56 (1.58-12.76) | 2.67 (1.88 – 9.05) | 2.32 (1.62 – 4.50) | 2.32 (1.37 – 3.60) | 3.23 (1.77-19.78) | 2.25 (1.45 – 6.60) | 3.00 (1.65-9.78) |
| Time from trauma to intervention (hours), median (IQR) | 4.88 (1.62 – 13.40) | 7.44 (3.02 – 16.44) | 6.78 (2.87 – 17.23) | 4.12 (2.93 – 11.17) | 5.75 (1.88 – 12.03) | 9.50 (2.50 – 18.75) | 3.33 (1.87 – 12.50) | 5.30 (3.57 – 11.83) | 2.00 (1.35 – 3.5) | 3.85 (2.06 – 12.63) | 1.58 (1.49 – 7.11) | 18.50 (5.98 – 30.52) | 15.41 (5.17 – 18.15) | 9.27 (5.13-11.90) |
| *Management* | | | | | | | | | | | | | | |
| Managed at university vs regional hospital alone, n (%) | 2972 (59%) / 2061 (41%) | 651 (52%)/599 (48%) | 898 (56%) / 778 (44%) | 182 (41%)/260 (59%) | 727 (65%) /461 (35%) | 159 (57%)/121 (43%) | 456 (64%) / 320 (36%) | 101 (56%)/80 (44%) | 436 (74%) / 163 (26%) | 77 (59%)/ 53 (41%) | 340 (66%) / 210 (34%) | 79 (62%)/49 (38%) | 115 (51%) / 129 (49%) | 53 (59%)/36 (41%) |
| Craniotomy (yes), n (%) | 587 (12%) | 129 (10%) | 206 (12%) | 37 (8%) | 143 (12%) | 29 (10%) | 109 (14%) | 23 (13%) | 51 (9%) | 11 (8%) | 59 (11%) | 18 (14%) | 19 (9%) | 11 (12%) |
| ICP-monitoring (yes), n (%) | 481 (10%) | 103 (8%) | 132 (8%) | 23 (5%) | 172 (14%) | 34 (12%) | 80 (10%) | 14 (8%) | 33 (5%) | 6 (5%) | 29 (5%) | 6 (5%) | 35 (14%) | 20 (22%) |
| Days on ventilator, median (IQR) | 2 (1-8) | 3 (1 - 7) | 3 (1-11) | 3 (1 - 8) | 2 (1-7) | 2 (1 - 7) | 2 (1-7) | 2 (1 - 5) | 2 (1-6) | 2 (1 - 4) | 2 (1-6) | 2 (1 - 7) | 5 (1-10) | 6 (2 - 10) |
| *Outcome* | | | | | | | | | | | | | | |
| Mortality, n (%) | 923 (18%) | 237 (19%) | 250 (15%) | 67 (15%) | 207 (17%) | 56 (20%) | 184 (24%) | 49 (27%) | 132 (22%) | 31 (24%) | 117 (21%) | 24 (19%) | 33 (13%) | 10 (11%) |
| **Missing data**: Age (n=0), Sex (n=0), Injury Mechanism (n=0), Management at university vs. Regional hospital alone (n= 1), GCS at admission (n=878), ASA (n=49), Mortality (n=106)  AIS = Abbreviated Injury Scale, ASA = American Society of Anesthesiologists scale. GCS = Glasgow Coma Scale. ICP = Intracranial Pressure, ISS = Injury Severity Score. IQR = Interquartile Range. | | | | | | | | | | | | | | |

**Supplementary Table 2. Variables regarding time from trauma to hospital, first computed tomography and intervention– entire cohort**

| **Variables** | Entire cohort | Stockholm | Middle | South | Western | Southeast | North | p-value |
| --- | --- | --- | --- | --- | --- | --- | --- | --- |
| Time from trauma to Hospital (hours), median (IQR) | 1.37 (0.85 – 6.44) | 1.23 (0.82 – 3.62) | 1.67 (0.90 – 9.82) | 1.28 (0.87 – 7.93) | 1.37 (0.92 – 8.42) | 1.40 (0.88 – 6.70) | 1.80 (0.78 – 7.50) | **<0.001** |
| Time from trauma to CT (hours), median (IQR) | 2.70 (1.58 – 9.45) | 2.80 (1.55 – 6.85) | 2.88 (1.70 – 12.37) | 2.48 (1.52 – 10.06) | 2.50 (1.63 – 9.04) | 2.63 (1.55 – 9.17) | 2.62 (1.48 – 9.33) | **0.002** |
| Time from trauma to intervention (hours), median (IQR) | 6.75 (2.95 – 15.33) | 4.33 (2.58 – 10.07) | 7.98 (2.70 – 15.90) | 7.78 (4.05 – 16.70) | 7.78 (4.05 – 16.70) | 7.78 (4.05 – 16.70) | 9.00 (6.42 – 13.88) | **<0.001** |
| **Missing data**: Time from trauma to hospital (n=0), Time from trauma to CT (n=208), Time from trauma to intervention (n=4055).  IQR = Interquartile Range. | | | | | | | | |

**Supplementary Table 3. Demography, trauma mechanism, injury severity, management, and outcome – managed at university hospital cohort**

| **Variables** | Entire cohort | Stockholm | Middle | South | Western | Southeast | North | p-value |
| --- | --- | --- | --- | --- | --- | --- | --- | --- |
| Patients, n (%) | 2972 (100%) | 898 (30%) | 727 (24%) | 456 (15%) | 436 (13%) | 340 (11%) | 115 (4%) | n/a |
| *Demography* | | | | | | | | |
| Age (years), median (IQR) | 59 (39 – 73) | 57 (38 – 72) | 60 (40 – 73) | 59 (40 – 73) | 62 (35 – 75) | 61 (39 – 76) | 58 (36 – 73) | ***0.011*** |
| Sex (male/female), n (%) | 2133/839 (72%/28%) | 642/256 (71%/29%) | 530/197 (73%/27%) | 321/135 (70%/30%) | 317/119 (73%/27%) | 237/103 (70%/30%) | 86/29 (75%/25%) | 0.801 |
| *Injury mechanism* | | | | | | | | ***<0.001*** |
| Fall, n (%) | 1675 (56%) | 537 (60%) | 372 (51%) | 249 (55%) | 255 (59%) | 202 (59%) | 60 (52%) |  |
| Road traffic accident, n (%) | 925 (31%) | 245 (27%) | 258 (36%) | 140 (31%) | 136 (31%) | 104 (31%) | 42 (37%) |  |
| Blunt, n (%) | 221 (7%) | 86 (10%) | 47 (6%) | 40 (9%) | 22 (5%) | 16 (5%) | 10 (9%) |  |
| Penetrating, n (%) | 39 (1%) | 14 (1%) | 8 (1%) | 4 (1%) | 8 (2%) | 5 (1%) | 0 (0%) |  |
| Explosion, n (%) | 5 (0%) | 2 (1%) | 1 (0%) | 2 (0%) | 0 (0%) | 0 (0%) | 0 (0%) |  |
| Other/unknown, n (%) | 107 (4%) | 14 (1%) | 41 (6%) | 21 (4%) | 15 (3%) | 13 (4%) | 3 (2%) |  |
| *Injury severity* | | | | | | | | |
| GCS at admission, median (IQR) | 14 (11 – 15) | 14 (11 – 15) | 14 (10 – 15) | 13 (10 – 15) | 14 (11 – 15) | 15 (12 – 15) | 13 (9 – 15) | ***<0.001*** |
| AIS head, median (IQR) | 3 (2 – 3) | 3 (2 – 3) | 3 (2 – 3) | 3 (2 – 3) | 3 (2 – 3) | 3 (2 – 4) | 3 (2 – 3) | ***<0.001*** |
| ISS, median (IQR) | 21 (14 – 26) | 21 (14 – 26) | 20 (14 – 26) | 22 (16 – 26) | 21 (13 – 27) | 21 (16 – 26) | 21 (16 – 26) | 0.689 |
| ASA score, median (IQR) | 2 (1 – 3) | 2 (1 – 3) | 2 (1 – 3) | 2 (1 – 2) | 2 (1 – 3) | 2 (1 – 3) | 2 (1 – 3) | **<0.001** |
| Epidural hematoma (yes), n (%) | 412 (14%) | 122 (14%) | 96 (13%) | 84 (18%) | 42 (10%) | 51 (15%) | 17 (15%) | ***0.009*** |
| Acute subdural hematoma (yes), n (%) | 2104 (71%) | 646 (72%) | 513 (71%) | 320 (70%) | 296 (68%) | 248 (73%) | 81 (70%) | 0.667 |
| Traumatic subarachnoid hemorrhage (yes), n (%) | 1555 (52%) | 518 (58%) | 351 (48%) | 245 (54%) | 219 (50%) | 147 (43%) | 75 (65%) | ***<0.001*** |
| Contusion, n (%) | 1652 (56%) | 568 (63%) | 348 (48%) | 274 (60%) | 243 (56%) | 160 (47%) | 59 (51%) | ***<0.001*** |
| *Logistics* | | | | | | | | |
| Time from trauma to Hospital (hours), median (IQR) | 1.48 (0.85 – 8.00) | 1.17 (0.78 – 4.44) | 2.00 (0.93 – 10.53) | 1.58 (0.92 – 9.00) | 1.34 (0.88 – 8.77) | 1.93 (0.97 – 12.08) | 2.25 (0.81 – 7.92) | ***<0.001*** |
| Time from trauma to CT (hours), median (IQR) | 2.47 (1.48 – 10.18) | 1.80 (1.27 – 5.93) | 2.95 (1.70 – 13.80) | 2.76 (1.53 – 11.94) | 2.44 (1.52 – 8.84) | 3.28 (1.71 – 15.66) | 3.20 (1.56 – 10.68) | ***<0.001*** |
| Time from trauma to intervention (hours), median (IQR) | 6.95 (3.15 – 15.50) | 4.32 (2.58 – 9.58) | 8.16 (2.87 – 16.58) | 8.15 (4.43 – 17.54) | 8.85 (3.50 – 21.63) | 12.12 (5.46 – 25.18) | 8.85 (6.52 – 11.50) | ***<0.001*** |
| *Management* | | | | | | | | |
| Craniotomy (yes), n (%) | 578 (19%) | 206 (23%) | 137 (19%) | 109 (24%) | 51 (12%) | 57 (17%) | 18 (16%) | ***<0.001*** |
| ICP-monitoring (yes), n (%) | 481 (16%) | 132 (15%) | 172 (24%) | 80 (18%) | 33 (7%) | 29 (9%) | 35 (30%) | ***<0.001*** |
| Days on ventilator, median (IQR) | 3 (1 – 9) | 4 (1 – 12) | 2 (1 – 8) | 2 (1 – 8) | 3 (1 – 7) | 3 (1 – 8) | 6 (2 – 13) | ***<0.001*** |
| *Outcome* | | | | | | | | |
| Mortality, n (%) | 471 (16%) | 148 (16%) | 93 (13%) | 81 (18%) | 92 (21%) | 48 (14%) | 9 (8%) | ***<0.001*** |
| **Missing data:** Age (n=0), Sex (n=0), Injury Mechanism (n=0), GCS at admission (n=741), ASA (n=46), Time from Trauma to CT (n=176), Time from Trauma to Intervention (n=2079), Mortality (n=68)  AIS = Abbreviated Injury Scale, ASA = American Society of Anesthesiologists scale. GCS = Glasgow Coma Scale. ICP = Intracranial Pressure, ISS = Injury Severity Score. IQR = Interquartile Range.  *This cohort includes both those who were immediately admitted at the university hospital and also those who were first admitted to a local hospital and immediately transferred to the university hospital. A total of 3 patients were excluded from both this and the local hospital’s cohort due to unknown transfer status.* | | | | | | | | |

**Supplementary Table 4. Demography, trauma mechanism, injury severity, management, and outcome – managed exclusively at local hospital cohort**

| **Variables** | Entire cohort | Stockholm | Middle | South | Western | Southeast | North | p-value |
| --- | --- | --- | --- | --- | --- | --- | --- | --- |
| Patients, n (%) | 2061 (100%) | 778 (38%) | 461 (22%) | 320 (16%) | 163 (8%) | 210 (10%) | 129 (6%) | n/a |
| *Demography* | | | | | | | | |
| Age (years), median (IQR) | 74 (57 – 84) | 76 (63 – 85) | 74 (55 – 83) | 74 (55 – 84) | 66 (50 – 78) | 73 (58 – 83) | 67 (47 – 80) | ***<0.001*** |
| Sex (male/female), n (%) | 1278/783 (62%/38%) | 437/341 (56%/44%) | 300/161 (65%/35%) | 213/107 (67%/33%) | 104/59 (64%/36%) | 130/80 (62%/38%) | 94/35 (73%/27%) | ***<0.001*** |
| *Injury mechanism* | | | | | | | | ***<0.001*** |
| Fall, n (%) | 1446 (70%) | 655 (84%) | 315 (68%) | 182 (57%) | 90 (55%) | 135 (65%) | 69 (53%) |  |
| Road traffic accident, n (%) | 493 (24%) | 84 (11%) | 120 (26%) | 119 (37%) | 58 (36%) | 63 (30%) | 49 (38%) |  |
| Blunt, n (%) | 80 (4%) | 32 (4%) | 16 (3%) | 12 (4%) | 6 (4%) | 5 (2%) | 9 (7%) |  |
| Penetrating, n (%) | 3 (0%) | 0(0%) | 1 (1%) | 0 (0%) | 0 (0%) | 2 (1%) | 0 (0%) |  |
| Explosion, n (%) | 1 (0%) | 0(0%) | 0 (0%) | 1 (0%) | 0 (0%) | 0 (0%) | 0 (0%) |  |
| Other/unknown, n (%) | 39 (2%) | 7 (1%) | 9 (2%) | 6 (2%) | 9 (5%) | 5 (2%) | 2 (2%) |  |
| *Injury severity* | | | | | | | | |
| GCS at admission, median (IQR) | 15 (13 – 15) | 15 (14 – 15) | 15 (12 – 15) | 15 (12 – 15) | 15 (13 – 15) | 14 (9 – 15) | 15 (14 – 15) | ***0.004*** |
| AIS head, median (IQR) | 3 (2 – 3) | 3 (2 – 3) | 3 (2 – 3) | 3 (2 – 3) | 3 (2 – 3) | 3 (2 – 3) | 3 (2 – 3) | ***<0.001*** |
| ISS, median (IQR) | 14 (10 – 21) | 13 (10 – 17) | 16 (10 – 22) | 17 (10 – 25) | 14 (10 – 22) | 18 (13 – 25) | 13 (9 – 19) | ***<0.001*** |
| ASA score, median (IQR) | 2 (2 – 3) | 3 (2 – 3) | 2 (2 – 3) | 2 (1 – 3) | 2 (1 – 2) | 2 (1 – 3) | 2 (1 – 3) | ***<0.001*** |
| Epidural hematoma (yes), n (%) | 113 (5%) | 29 (4%) | 18 (4%) | 30 (9%) | 11 (7%) | 12 (6%) | 13 (10%) | ***<0.001*** |
| Acute subdural hematoma (yes), n (%) | 1546 (75%) | 630 (81%) | 341 (74%) | 225 (70%) | 113 (69%) | 150 (71%) | 87 (67%) | ***<0.001*** |
| Traumatic subarachnoid hemorrhage (yes), n (%) | 860 (42%) | 327 (42%) | 195 (42%) | 137 (43%) | 60 (37%) | 77 (37%) | 64 (50%) | 0.206 |
| Contusion, n (%) | 822 (40%) | 306 (39%) | 185 (40%) | 137 (43%) | 66 (40%) | 79 (38%) | 49 (38%) | 0.218 |
| *Logistics* | | | | | | | | |
| Time from trauma to Hospital (hours), median (IQR) | 1.30 (0.85 – 3.43) | 1.28 (0.86 – 2.81) | 1.38 (0.85 – 8.52) | 1.16 (0.78 – 4.09) | 1.43 (1.00 – 4.60) | 1.13 (0.78 – 1.83) | 1.62 (0.78 – 6.00) | ***<0.001*** |
| Time from trauma to CT (hours), median (IQR) | 2.95 (1.78 – 7.00) | 4.11 (2.18 – 7.47) | 2.84 (1.71 – 10.02) | 2.40 (1.50 – 6.27) | 2.67 (1.88 – 9.05) | 2.32 ( 1.37 – 3.60) | 2.25 (1.45 – 6.60) | ***<0.001*** |
| Time from trauma to intervention (hours), median (IQR) | 4.88 (1.62 – 13.40) | 6.78 (2.87 – 17.23) | 5.75 (1.88 – 12.03) | 3.33 (1.87 – 12.50) | 2.00 (1.35 – 3.5) | 1.58 ( 1.49 – 7.11) | 15.41 (5.17 – 18.15) | 0.209 |
| *Management* | | | | | | | | |
| Craniotomy (yes), n (%) | 9 (0.4%) | 0 (0%) | 6 (1.3%) | 0 (0%) | 0 (0%) | 2 (1%) | 1 (1%) | ***0.010*** |
| ICP-monitoring (yes), n (%) | 0 (0%) | 0 (0%) | 0 (0%) | 0 (0%) | 0 (0%) | 0 (0%) | (0%) | n/a |
| Days on ventilator, median (IQR) | 1 (1 – 3) | 2 (1 – 4) | 2 (1 – 3) | 1 (1 – 2) | 1 (1 – 2) | 1 (1 – 2) | 1 (1 – 1) | ***<0.001*** |
| *Outcome* | | | | | | | | |
| Mortality, n (%) | 452 (22%) | 102 (13%) | 114 (25%) | 103 (32%) | 40 (25%) | 69 (33%) | 24 (19%) | ***<0.001*** |
| **Missing data:** Age (n=0), Sex (n=0), GCS at admission (n=135), ASA (n=10), Time from Trauma to CT (n=30), Time from Trauma to Intervention (n=1973), Mortality (n=5).  AIS = Abbreviated Injury Scale, ASA = American Society of Anesthesiologists scale. GCS = Glasgow Coma Scale. ICP = Intracranial Pressure, ISS = Injury Severity Score. IQR = Interquartile Range.  *This cohort includes patients managed exclusively at a local hospital. A total of 3 patients were excluded from both this and the university hospital’s cohort due to unknown transfer status.* | | | | | | | | |
